# The *Vibrio cholerae* VexGH RND Efflux System Maintains Cellular Homeostasis by Effluxing Vibriobactin

**DOI:** 10.1128/mBio.00126-17

**Published:** 2017-05-16

**Authors:** Dillon E. Kunkle, X. Renee Bina, James E. Bina

**Affiliations:** Department of Microbiology and Molecular Genetics, University of Pittsburgh School of Medicine, Pittsburgh, Pennsylvania, USA; Harvard Medical School

## Abstract

Resistance-nodulation-division (RND) superfamily efflux systems have been widely studied for their role in antibiotic resistance, but their native biological functions remain poorly understood. We previously showed that loss of RND-mediated efflux in *Vibrio cholerae* resulted in activation of the Cpx two-component regulatory system, which mediates adaptation to stress resulting from misfolded membrane proteins. Here, we investigated the mechanism linking RND-mediated efflux to the Cpx response. We performed transposon mutagenesis screening of RND-deficient *V. cholerae* to identify Cpx suppressors. Suppressor mutations mapped to genes involved in the biosynthesis of the catechol siderophore vibriobactin. We subsequently demonstrated that vibriobactin secretion is impaired in mutants lacking the VexGH RND efflux system and that impaired vibriobactin secretion is responsible for Cpx system activation, suggesting that VexGH secretes vibriobactin. This conclusion was bolstered by results showing that *vexGH* expression is induced by iron limitation and that *vexH*-deficient cells exhibit reduced fitness during growth under iron-limiting conditions. Our results support a model where VexGH contributes to cellular homeostasis by effluxing vibriobactin. In the absence of *vexGH*, retained vibriobactin appears to chelate iron from iron-rich components of the respiratory chain, with the deferrated proteins functioning to activate the Cpx response. Our collective results demonstrate that a native function of the *V. cholerae* VexGH RND efflux system is in vibriobactin secretion and that vibriobactin efflux is critical for maintenance of cellular homeostasis.

## IMPORTANCE

RND efflux systems are ubiquitous Gram-negative transporters that play critical roles in antimicrobial resistance. In addition to antimicrobial resistance, RND transporters also affect the expression of diverse phenotypes, including virulence, cell metabolism, and stress responses. The latter observations suggest that RND transporters fulfill unknown physiological functions in the cell independently of their role in antimicrobial resistance. *Vibrio cholerae* is representative of many Gram-negative bacteria in encoding multiple RND transporters that are redundant in antimicrobial resistance and affect multiple phenotypes. Here we describe a novel function of the *V. cholerae* VexGH RND transporter in vibriobactin secretion. We show that vibriobactin production in VexGH-deficient cells impacts cell homeostasis, leading to activation of the Cpx stress response and reduced fitness under iron-limiting conditions. Our results highlight a native physiological function of an RND transporter and provide insight into the selective forces that maintain what was thought to be a redundant multidrug transporter.

## INTRODUCTION

*Vibrio cholerae* is a Gram-negative bacterium and the causative agent of the life-threatening diarrheal disease cholera. *V. cholerae* is a native inhabitant of aquatic environments from which humans acquire cholera through the ingestion of *V. cholerae*-contaminated food or water (1, 2). Following ingestion, *V. cholerae* colonizes the small intestine, where it produces a variety of virulence factors that result in the production of a severe secretory diarrhea that is the hallmark of the disease cholera (2).

The ability of *V. cholerae* to colonize and replicate in the human gastrointestinal (GI) tract is dependent upon its ability to adapt to its environment. This includes overcoming colonization barriers provided by the presence of toxic antimicrobial compounds such as bile salts, fatty acids, and products of the innate immune system. Many of these compounds also serve as environmental cues that activate the expression of adaptive responses in *V. cholerae* that facilitate survival and replication in the GI tract. One component of these adaptive responses is enhancement of antimicrobial resistance. This is accomplished by a multifactorial response that includes the expression of active efflux systems, reduced outer membrane permeability, and the expression of stress response systems that mitigate cellular damage resulting from exposure to toxic molecules (3).

Active efflux systems belonging to the resistance-nodulation-division (RND) superfamily are critical for intrinsic and induced antimicrobial resistance in Gram-negative bacteria, including *V. cholerae* (3). The RND efflux systems are ubiquitous tripartite transporters that exhibit a broad substrate specificity that includes antibiotics, detergents, antimicrobial peptides, and dyes (4). For this reason, RND transporters play a critical role in the evolution of multiple-antibiotic-resistant bacteria. However, the native function of the RND efflux systems in most bacteria is poorly understood. Numerous studies across multiple genera have linked RND transporters to the expression of diverse phenotypes, suggesting that their function in bacterial biology extends beyond their well-established role in antimicrobial resistance (5). However, the mechanisms by which RND transporters contribute to most of these phenotypes are not known.

The *V. cholerae* genome encodes six RND efflux systems. In addition to mediating resistance to antimicrobial compounds, the *V. cholerae* RND efflux systems are also required for virulence gene expression and colonization of the infant mouse (6–8). We recently showed that two *V. cholerae* RND multidrug efflux systems (i.e., *vexAB* and *vexGH*) and the Cpx envelope stress response were reciprocally regulated (9). The Cpx system is a two-component system that regulates adaptive responses to perturbations that generate misfolded envelope proteins (10). Environmental stimuli that activate the *V. cholerae* Cpx response include high salinity, iron stress, proteins containing aberrant disulfide bonds, and loss of RND-mediated efflux (9–11). We found that mutation of *vexAB* and *vexGH* resulted in constitutive activation of the Cpx system and that activation of the Cpx system resulted in the upregulation of *vexAB* and *vexGH*. While the molecular mechanism involved in the reciprocal regulation of the RND transporters and the Cpx system is unknown, the genetic linkage between the Cpx response and the expression of these two broad-spectrum RND transporters indicates that *V. cholerae* employs a multifaceted strategy to alleviate extracytoplasmic stress by activating efflux to remove deleterious molecules from the cell while mitigating cellular damage via the Cpx response.

In this study, we sought to define the molecular mechanisms linking RND-mediated efflux to the activation of the *V. cholerae* Cpx system. To identify genes involved in this process, we performed a transposon mutagenesis screen to identify suppressors of the Cpx system in a RND-deficient *V. cholerae* mutant. The results of this screen identified several suppressors that mapped to genes involved in biosynthesis of the catechol siderophore vibriobactin. Subsequent analyses showed that vibriobactin secretion was impaired in RND-deficient *V. cholerae* and that the inability of the RND mutants to secrete vibriobactin resulted in activation of the Cpx system. These findings supported the novel conclusion that the RND efflux systems function in vibriobactin secretion. We further found that *vexGH* expression was regulated by iron and that VexGH directly contributed to vibriobactin secretion, suggesting that a native function of VexGH is to efflux vibriobactin. The intracellular accumulation of vibriobactin in mutants lacking *vexGH* appeared to be directly responsible for the activation of the Cpx system. Further, this vibriobactin-dependent activation of the Cpx system was dependent on aerobic respiration and succinate dehydrogenase; suggesting that retained vibriobactin directly impairs the function of iron-rich components of the respiratory chain. The inability to efficiently secrete vibriobactin in RND-deficient cells led to attenuated growth under iron-limiting conditions. Our collective studies demonstrate a physiological function of a *V. cholerae* RND efflux transporter in iron acquisition and the maintenance of cellular homeostasis.

## RESULTS

### Identification of Cpx suppressors in RND-negative *V. cholerae*.

We recently found that mutation of the *V. cholerae* RND family transporters resulted in constitutive expression of the Cpx system (9, 10). To elucidate the mechanism behind this phenotype, we performed a transposon mutagenesis screen to identify suppressors of the Cpx system in RND-negative strain JB485. We generated a transposon library in JB485 bearing a chromosomal *cpxP-lacZ* reporter and screened ~10,000 mutants on lysogeny broth (LB) 5-bromo-4-chloro-3-indolyl-β-d-galactopyranoside (X-Gal) agar plates for white colonies; *cpxP* is positively regulated by CpxR and serves as a reporter of the activation state of the Cpx system (9–11). The transposon screen resulted in the identification of six transposon mutants that contained insertions in four independent genes.

Three of the six transposon insertions mapped to vibriobactin biosynthesis genes *vibF* (two hits) and *vibD* (1 hit). Vibriobactin is a catechol siderophore utilized for iron acquisition and is the only known siderophore produced by *V. cholerae* (12). Vibriobactin is produced by a sequential biosynthetic pathway that consists of the products of the *vibABCDEFGH* genes (see Fig. S1 in the supplemental material). Strains containing mutations in any of the *vib* genes do not produce vibriobactin (13). Thus, the finding that *vibD* and *vibF* insertions suppressed the Cpx system suggested that vibriobactin contributed to induction of the Cpx system in JB485. Two hits mapped to *sdhA*, which encodes a subunit of succinate dehydrogenase (i.e., complex II). Succinate dehydrogenase is an iron-sulfur protein that catalyzes the oxidation of succinate to fumarate in the electron transport chain (ETC) and the Krebs cycle (14). One insertion mapped to *epsK*, which encodes a component of the type II secretion system. Interestingly, all four of the suppressor genes are regulated in response to iron (15, 16), suggesting that RND efflux-dependent activation of the Cpx system might be linked to vibriobactin production and/or iron homeostasis.

10.1128/mBio.00126-17.1 FIG S1  The vibriobactin biosynthesis pathway. Vibriobactin is composed of three molecules of dihydroxybenzoate (shown in black) attached to a norsperimidine backbone (blue). One dihydroxybenzoate molecule is attached directly to the norspermidine backbone, and the other two are attached through cyclized threonine linkers (red). This image was adapted from a previously published report (S. M. Payne, A. R. Mey, and E. E. Wyckoff, Microbiol Mol Biol Rev 80:69−90, 2016; doi:10.1128/MMBR.00046-15). Download FIG S1, EPS file, 0.2 MB. Copyright © 2017 Kunkle et al. 2017 Kunkle et al. This content is distributed under the terms of the Creative Commons Attribution 4.0 International license.

### Vibriobactin is responsible for Cpx activation in strains lacking RND-mediated efflux.

**FIG 1:**
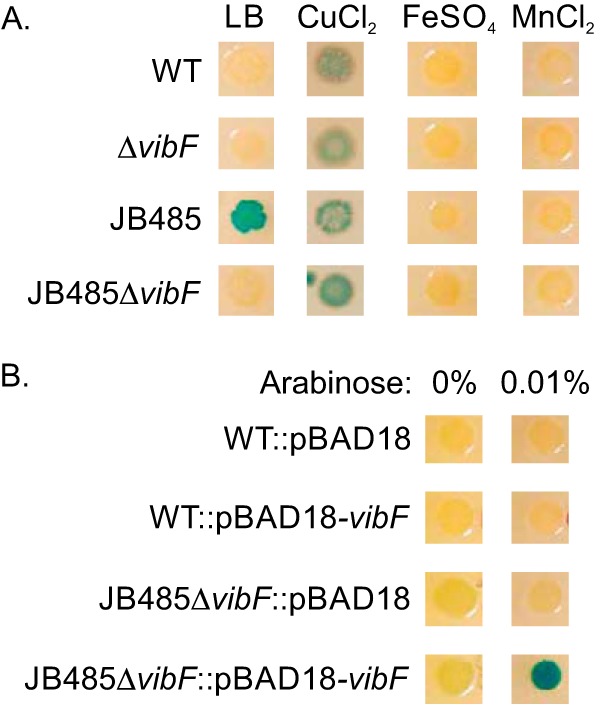
Expression of the Cpx system in RND efflux-deficient *V. cholerae* is dependent upon vibriobactin production. Strains harboring a chromosomal *cpxP-lacZ* reporter were inoculated onto the surfaces of LB X-Gal agar plates and incubated overnight at 37°C before being photographed. (A) Effects of FeSO_4_, CuCl_2_, and MnCl_2_ on *cpxP-lacZ* expression in the *V. cholerae* strains indicated. All three compounds were added to the LB agar at a final concentration of 500 μM. (B) Effect of ectopic *vibF* expression on *cpxP-lacZ* expression in the WT and JB485Δ*vibF* strains. The WT and JB485Δ*vibF* strains harboring the expression plasmids indicated following growth on LB X-Gal agar plates containing 0 or 0.01% arabinose.

The above described screening implicated vibriobactin production in the activation of the Cpx system in JB485. To confirm this, we constructed a clean *vibF* deletion in wild-type (WT) strain JB58 and mutant strain JB485. We then introduced the chromosomal *cpxP-lacZ* reporter into each of the respective strains before assessing the activation status of the Cpx system on LB X-Gal agar plates. The results showed that JB58 and its isogenic Δ*vibF* mutant produced white colonies on LB X-Gal agar (Fig. 1A), indicating that the Cpx system was inactive. RND-negative strain JB485 produced blue colonies on LB X-Gal agar, confirming constitutive Cpx activation as previously reported (9). In contrast, the JB485Δ*vibF* mutant produced white colonies on the same agar, confirming the results of our transposon screening. As a control, we examined the test strains on LB X-Gal agar containing CuCl_2_, a documented inducer of the Cpx system (10). All of the test strains produced blue colonies in the presence of CuCl_2_, confirming that the Cpx system was functional in each strain.

We further verified that *vibF* was responsible for Cpx induction in JB485 by performing *vibF* complementation studies. We cloned *vibF* under control of the arabinose-regulated promoter in pBAD18 and transformed the resulting plasmid (i.e., pBAD18-*vibF*) and the empty-vector control into the WT and JB485Δ*vibF* strains bearing a chromosomal *cpxP-lacZ* reporter. We then examined the activation state of the Cpx system in the presence or absence of arabinose on LB X-Gal plates. The results revealed that all of the strains produced white colonies in the absence of arabinose (Fig. 1B). Growth of the strains in the presence of arabinose demonstrated that ectopic *vibF* expression in JB485Δ*vibF* activated *cpxP-lacZ* expression, as indicated by the production of blue colonies. This confirmed that *vibF* is required for Cpx activation in the absence of RND-mediated efflux. These results supported the conclusion that induction of the Cpx system in cells lacking RND-mediated efflux is dependent on vibriobactin production.

Vibriobactin biosynthesis is negatively regulated by the master iron regulator Fur. On the basis of this fact, we hypothesized that if induction of the Cpx system in JB485 is a result of vibriobactin production, then addition of iron to the growth medium would repress vibriobactin production and suppress the Cpx system. To test this, we cultured the WT, JB485, and isogenic Δ*vibF* mutant strains bearing the *cpxP-lacZ* reporter on LB X-Gal plates containing 500 μM FeSO_4_. The results revealed that FeSO_4_ suppressed *cpxP-lacZ* expression in JB485, confirming our hypothesis (Fig. 1A). Manganese can also complex with Fur to repress Fur-regulated genes (17, 18). We therefore repeated these experiments with LB X-Gal plates containing 500 μM MnCl_2_. The results revealed that manganese also suppressed the Cpx system in JB485 (Fig. 1A). Together, these results were consistent with the notion that vibriobactin production was responsible for activation of the Cpx system in *V. cholerae* cells that lacked RND-mediated efflux.

### JB485 culture supernatants contain reduced amounts of siderophore.

The above-described results indicated that vibriobactin production is required for activation of the Cpx system in RND efflux-deficient *V. cholerae*. Vibriobactin is produced in the cytoplasm before being secreted by an unknown mechanism. On the basis of this, we hypothesized that the *V. cholerae* RND efflux systems functioned in vibriobactin secretion and that activation of the Cpx system in JB485 might have resulted from intracellular vibriobactin accumulation due to the absence of RND-mediated efflux. If this was true, we posited that JB485 culture supernatants should contain reduced amounts of iron-chelating compounds than the WT. To test this, we quantified siderophore secretion in the WT and JB485 strains by using the chrome azurol S (CAS) assay. We used a *vibC* mutant as a negative control in these studies because VibC catalyzes the first step in vibriobactin biosynthesis (see Fig. S1). This circumvents potential problems associated with downstream *vib* mutations (e.g., *vibF*) that may accumulate biosynthetic intermediates that could influence the CAS assay. The CAS assay is based on the formation of a chromogenic complex made up of CAS (20), hexadecyltrimethylammonium bromide, and Fe^3+^ that can be quantified spectrophotometrically. The presence of strong iron chelators like siderophores titrates Fe^3+^ from the dye complex, resulting in decreased absorbance at 630 nm. Thus, the absorbance is inversely proportional to the amount of iron-chelating agents present in the culture supernatant (20).

**FIG 2:**
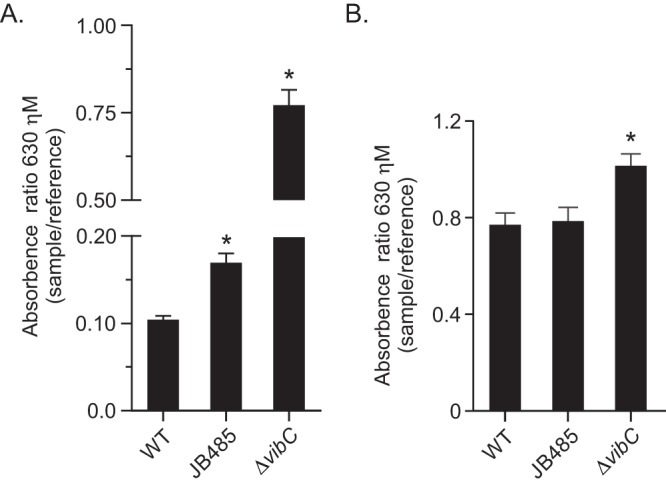
Culture supernatants from RND efflux-deficient *V. cholerae* contain reduced siderophore concentrations. The CAS assay was used to quantify siderophore production in cell-free culture supernatants (A) and whole-cell lysates (B) of the strains indicated following growth to saturation in T medium without iron supplementation. The data are presented as the average ± the standard deviation of three independent experiments. *, *P* < 0.05.

We cultured the WT, JB485, and Δ*vibC* mutant strains in minimal T medium without iron supplementation to equivalent optical densities (ODs), growth conditions demonstrated to induce *V. cholerae* siderophore production (21). Cell-free supernatants from the resulting cultures were then assessed with the CAS assay. The results showed a higher absorbance ratio in JB485 than in the WT, indicating that the JB485 supernatants contained lower amounts of siderophore than the WT supernatant (Fig. 2A). In contrast, a *vibC* mutant showed the greatest increase in the absorbance ratio, which was consistent with its defect in vibriobactin production. Analysis of whole-cell lysates showed that there was no difference in total siderophore production between the WT and JB485, while siderophore production in the Δ*vibC* mutant was decreased (Fig. 2B). Together, these data demonstrated that siderophore secretion was impaired in JB485 and suggested that the RND transporters functioned in siderophore secretion. However, the fact that the *vibC* supernatants contained lower amounts of siderophore than the RND-deficient cells suggests that other mechanisms must also contribute to siderophore export.

### Vibriobactin secretion is impaired in JB485.

The CAS assay is nonspecific and does not discriminate between specific siderophores (i.e., vibriobactin) and other iron-binding compounds. We therefore performed growth stimulation cross-feeding assays to determine if the secreted iron-binding compound observed as described above was vibriobactin (22, 23). The cross-feeding assays assess the ability of a vibriobactin-producing test strain to stimulate the growth of a vibriobactin-negative indicator strain in iron-limiting agar. The indicator strain in our assays was JB58 Δ*vibC*. The *vibC* mutant cannot produce vibriobactin but retains the ability to use exogenously supplied vibriobactin (23).

**FIG 3:**
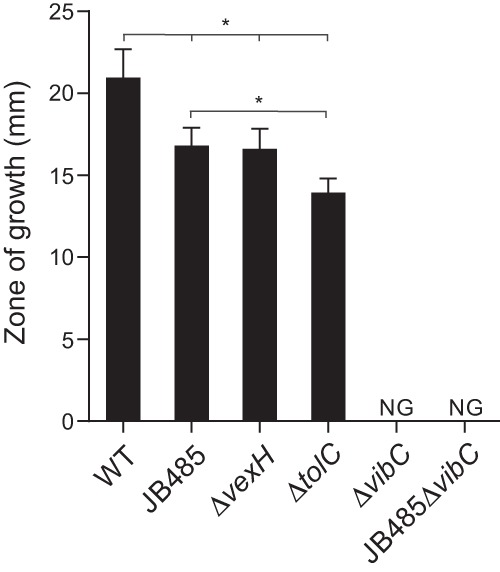
Vibriobactin secretion is impaired in *V. cholerae* lacking RND-mediated efflux. The *V. cholerae* strains indicated were examined with the cross-feeding growth stimulation assay. The producer strains were spotted onto the surface of solidified LB agar plates containing 270 μM dipyridyl and the Δ*vibC* mutant indicator strain before the plates were incubated at 37°C. The diameter of the zone of stimulated growth of the Δ*vibC* mutant indicator strain was then measured after 24 h. The data are presented as the average ± the standard deviation of three independent experiments performed in triplicate. *, *P* < 0.05. NG, no growth.

The results of the cross-feeding assays showed that the WT strongly stimulated the growth of the indicator strain, whereas an isogenic Δ*vibC* mutant failed to stimulate growth (Fig. 3). This indicated that transcomplementation of the Δ*vibC* mutant indicator strain was dependent upon vibriobactin production by the test strains. JB485 showed a lesser ability to stimulate the growth of the indicator strain than the WT (Fig. 3). This finding was consistent with the CAS assay and further supported the conclusion that JB485 was impaired in vibriobactin secretion. Deletion of *vibC* in JB485 completely blocked its ability to stimulate the growth of the Δ*vibC* mutant indicator strain. This suggested that the growth-stimulatory compound secreted by JB485 was vibriobactin. These results, combined with the CAS assay results, strongly suggest that vibriobactin secretion is impaired in JB485 and support the novel conclusion the *V. cholerae* RND transporters function in vibriobactin secretion.

The expression of the *vexGH* RND efflux system has been reported to be under the influence of both the Cpx system and Fur (9, 24). We found that activation of the Cpx system resulted in *vexGH* upregulation via CpxR, while *vexGH* mutation resulted in activation of the Cpx system (9). A separate study showed that the *vexGH* promoter contains a Fur box and is regulated in response to iron availability (11). On the basis of these findings and our above-described data, we hypothesized that VexGH is directly involved in vibriobactin secretion. To test this, we examined a *vexH* mutant in the cross-feeding bioassay. The results showed that the Δ*vexH* mutant phenocopied JB485 for growth stimulation of the indicator strain, confirming that VexGH functions in vibriobactin secretion (Fig. 3).

There was a large difference between the levels of growth stimulation observed in the RND mutant and the Δ*vibC* mutant (Fig. 3). This indicated that vibriobactin can be secreted by additional mechanisms besides RND-mediated efflux. To determine if other active efflux systems contribute to vibriobactin export, we examined a *tolC* mutant. TolC serves as the outer membrane pore protein for many different transport systems, including the RND family, major facilitator family, and ATP binding cassette transporters (25). The expression of *tolC* is also influenced by CpxR (9, 11). The Δ*tolC* mutant was slightly less able to stimulate indicator strain growth than the RND mutant (Fig. 3). This suggests that other active transport systems likely contribute to vibriobactin secretion. However, the fact that *tolC* deletion did not completely abrogate growth stimulation indicates that vibriobactin can escape from the cell in the absence of TolC-dependent active efflux. It is unknown how this occurs, but it is possible that vibriobactin could escape through porin channels. The fact that the *ompT* porin is regulated by Fur supports this idea (26). Taken together, the cross-feeding results suggested that the RND efflux systems, and VexGH in particular, function in vibriobactin secretion.

### *vexGH* regulation by iron is independent of the Cpx system.

**FIG 4:**
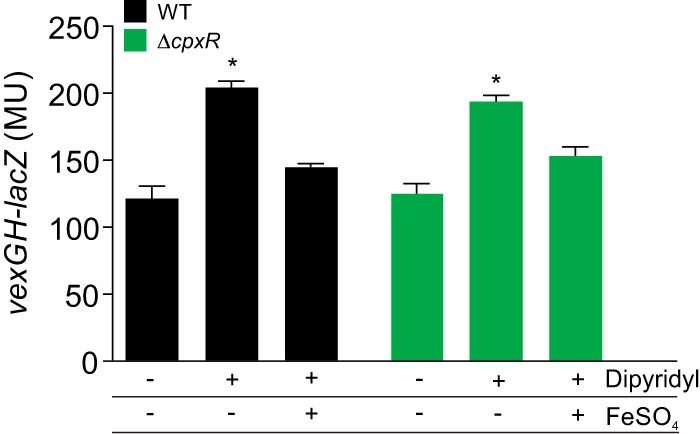
Iron-dependent regulation of *vexGH* is independent of the Cpx system. The WT and an isogenic Δ*cpxR* mutant were cultured in LB broth in the presence or absence of 130 μM dipyridyl or 130 μM dipyridyl plus 130 μM FeSO_4_. After 3 h of growth at 37°C with shaking, triplicate aliquots were assayed for β-galactosidase activity. The data are presented as the average ± the standard deviation of three independent experiments. *, *P* < 0.05. MU, Miller units.

The above-described results indicated that the RND transporters are involved in iron acquisition. The expression of many RND efflux systems are regulated in response to environmental cues and/or by their respective efflux substrates (9, 27). Thus, we hypothesized that if any of the RND transporters are involved in iron acquisition then their expression would be regulated in response to iron. We tested this by assaying for iron-dependent changes in the expression of each of the six *V. cholerae* RND systems following growth in LB broth plus or minus the iron-chelating chemical dipyridyl. The results revealed that dipyridyl increased *vexGH* expression (Fig. 4) but did not significantly affect the expression of the other five RND systems (data not shown). The addition of an equimolar amount of FeSO_4_ to the dipyridyl cultures returned *vexGH* expression to a level that was equivalent to that of cells grown in the absence of dipyridyl (Fig. 4), confirming that increased *vexGH* expression was a result of iron limitation and not due to a nonspecific effect of dipyridyl. These results indicated that *vexGH* is regulated in response to iron, a conclusion that is consistent with a report linking *vexGH* to the Fur regulon (24).

We previously reported that *vexGH* was positively regulated by the Cpx system (9), while others reported that the Cpx system was activated in response to iron limitation during growth in the presence of dipyridyl on LB agar lacking NaCl (11). This suggested the possibility that the Cpx system was responsible for *vexGH* activation under the iron-limiting conditions described above. To address this possibility, we repeated the above-described experiments with a Δ*cpxR* mutant. The Δ*cpxR* mutant mirrored the WT results (Fig. 4). The fact that *vexGH* was induced to similar levels under iron-limited conditions in both the WT and the Δ*cpxR* mutant indicated that the iron-dependent regulation of *vexGH* transcription occurred by a *cpxR*-independent mechanism. We presume that the iron-dependent regulation occurred by a Fur-dependent process as previously reported (24), but additional work is required to confirm this.

### RND-deficient mutants are not iron stressed.

**FIG 5:**
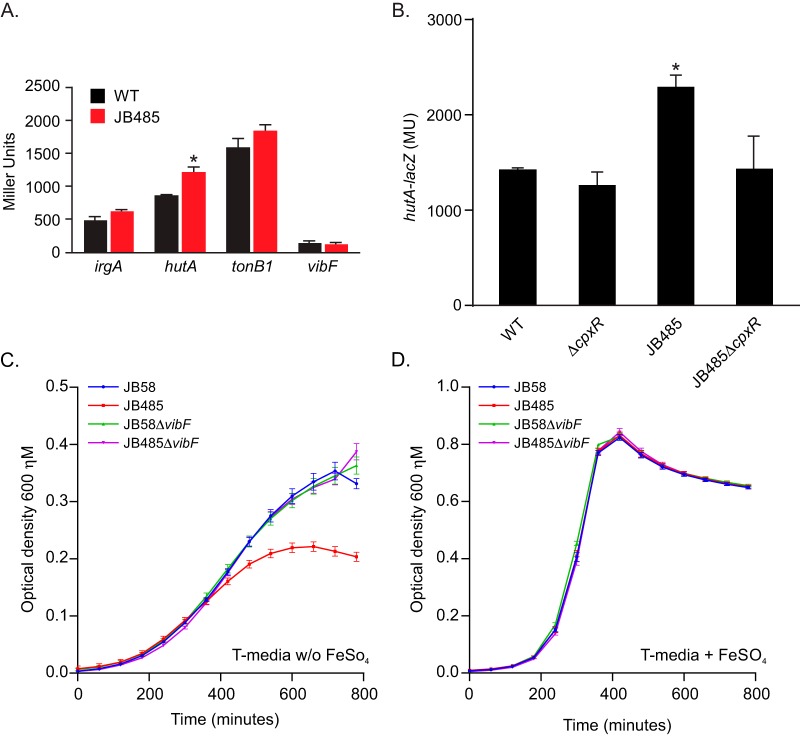
Loss of RND-mediated efflux does not affect the expression of Fur-regulated genes. (A) WT strain JB58 or the RND-negative mutant JB485 bearing transcriptional reporters for the Fur-regulated genes indicated were cultured in LB broth to the mid-logarithmic phase, and then the expression of the reporters was assessed by the β-galactosidase assay. *, *P* < 0.05 relative to the WT. (B) Iron-dependent upregulation of *hutA* is dependent on CpxR. The *V. cholerae* strains indicated bearing a *hutA-lacZ* transcriptional reporter were cultured as described for panel A. *, *P* < 0.05 relative to the WT. MU, Miller units. (C) RND-deficient *V. cholerae* has impaired fitness in iron-depleted medium. Overnight T medium cultures of the strains indicated were diluted 1:100 in fresh T medium without (C) or with (D) FeSO_4_ supplementation and cultured at 37°C with shaking in a microtiter plate reader. Cell growth was recorded every 30 min as the OD_600_. The data are presented as the average ± the standard error of the mean of three independent experiments.

We hypothesized that the defect in vibriobactin secretion in JB485 may result in a reduced ability to obtain iron. In *V. cholerae*, as in many bacteria, the control of iron homeostasis is mediated by the ferric uptake regulator (Fur), which regulates the expression of iron acquisition genes in response to iron availability (24, 28, 29). Fur functions primarily as a repressor. Under iron-replete conditions Fur binds Fe^2+^, which enables binding to conserved DNA sequences (i.e., Fur box) in the promoters of target genes to repress their expression. Under iron-depleted conditions, the Fe^2+^ binding equilibrium is shifted and iron is released from Fur, resulting in the derepression of target genes (30, 31). On the basis of this, we postulated that if the RND-negative mutant was iron stressed, then the expression of Fur-regulated genes would increase relative to that in the WT. We therefore compared the expression levels of four Fur-regulated genes (*irgA*, *hutA*, *tonB*, and *vibF*) in the WT and JB485 strains (24, 28). The results revealed no significant difference in *irgA*, *tonB1*, or *vibF* expression between the WT and JB485 (Fig. 5A). In contrast, *hutA* expression was higher in JB485 than in the WT. We previously showed that *hutA* was one of the most highly upregulated genes in the *V. cholerae* Cpx regulon (9); suggesting that *hutA* expression, like *vexGH* expression, is regulated by both Fur and the Cpx system. It is noteworthy that CpxR has also been linked to the expression of some iron acquisition genes in *V. cholerae* (11). To determine if increased *hutA* expression in JB485 was due to an iron acquisition defect or due to the Cpx system, we compared *hutA* expression in the WT, a Δ*cpxR* mutant, JB485, and JB485Δ*cpxR* during growth in LB broth. The results showed that deletion of *cpxR* in the WT did not affect basal-level *hutA* expression but that *cpxR* deletion in JB485 reduced *hutA* expression to WT levels (Fig. 5B). This confirmed that the increased *hutA* expression in JB485 was due to CpxR and not due to an iron acquisition defect. From these results, we concluded that loss of RND-mediated efflux did not result in an iron acquisition defect under the conditions tested.

The above-described conclusion was further validated by comparing the growth of the WT, Δ*vibF* mutant, JB485, and JB485Δ*vibF* strains in iron-limited T medium. The results revealed that the WT and the Δ*vibF* mutant exhibited equivalent growth in iron-limited T medium (Fig. 5C). Since *vibF* is essential for vibriobactin production, these results demonstrate that vibriobactin is dispensable for *V. cholerae* growth in iron-limited T medium. In contrast JB485 exhibited impaired growth in iron-limited T medium, whereas an isogenic JB485Δ*vibF* mutant exhibited WT growth kinetics (Fig. 5C); suggesting that the inability to efflux vibriobactin was deleterious to growth under iron-limiting conditions. Control experiments in iron-sufficient T medium confirmed that JB485 did not exhibit a nonspecific growth defect in T medium (Fig. 5D). Collectively, these results suggested that RND-mediated efflux is dispensable for growth under iron-sufficient conditions but enhances fitness during growth under iron-limiting conditions. The decreased fitness of the RND-negative mutant under iron-limiting conditions was likely due to the combined effects of increased vibriobactin production and a defect in vibriobactin efflux.

### Oxidative stress activates the Cpx response in *V. cholerae*.

**FIG 6:**
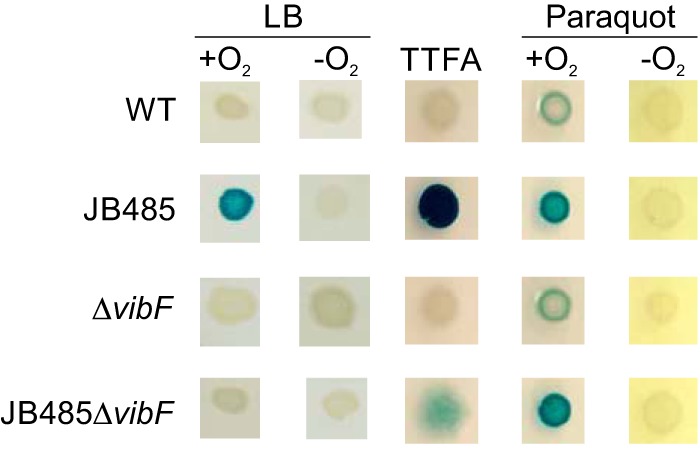
ROS activate the *V. cholerae* Cpx system. The *V. cholerae* strains indicated bearing a chromosomal *cpxP-lacZ* reporter were inoculated onto the surfaces of LB X-Gal agar plates or plates containing the complex II inhibitor TTFA at 2.5 μM or the superoxide-producing drug paraquat at 500 μM. The plates were incubated overnight at 37°C in the presence or absence of oxygen before being photographed. Anaerobic conditions were achieved with a BD GasPak EZ Pouch System.

Our collective data suggest that an inability to secrete vibriobactin results in activation of the Cpx system. However, the mechanism by which retained vibriobactin activated the Cpx system was unclear. The fact that we identified *sdhA* as a Cpx suppressor in JB485 suggested that aerobic respiration is required to generate the Cpx-activating signal. SdhA is part of complex II (i.e., succinate dehydrogenase) of the ETC (14, 32). Reactive oxygen species (ROS) are produced by the ETC as a byproduct of aerobic respiration. ROS can oxidize proteins and result in the formation of aberrant disulfide bonds and misfolded proteins, which can serve as activating signals for the Cpx system (10). If aerobic respiration is required for activation of the Cpx system in JB485, then growth under anaerobic conditions should suppress Cpx expression in JB485. We tested this by culturing JB485 *cpxP-lacZ* on LB X-Gal agar under anaerobic conditions. The results confirmed our hypothesis and showed that anaerobic growth completely suppressed the *cpxP-lacZ* reporter in JB485 (Fig. 6).

We next tested if ROS alone could induce the Cpx response. This was done by culturing the *cpxP-lacZ* reporter strains on agar plates containing paraquat. Paraquat is an oxidative-stress-inducing agent that catalyzes superoxide formation (33). Growth on paraquat activated the Cpx system in the WT, Δ*vibF* mutant, JB485, and JB485Δ*vibF* strains under aerobic conditions but not under anaerobic growth conditions (Fig. 6). These findings confirmed that oxidative stress generated by paraquat was sufficient to activate the *V. cholerae* Cpx system. We performed similar experiments with hydrogen peroxide. However, hydrogen peroxide did not activate the Cpx system, which was consistent with a previous report (11). These divergent results may stem from the fact that paraquat was reported to catalyze superoxide production in *V. cholerae*, whereas hydrogen peroxide abrogated superoxide production (34). From these results, we concluded that ROS can activate the Cpx response in *V. cholerae*.

The fact that Cpx suppressors in JB485 mapped to respiratory complex II suggested that complex II may be directly responsible for generating the Cpx-activating signal. We therefore tested if chemical inhibition of complex II in the WT or JB485 affects Cpx activation. To do this, we inoculated the WT, Δ*vibF* mutant, JB485, and JB485Δ*vibF cpxP-lacZ* reporter strains onto LB X-Gal agar containing thenoyltrifluoroacetone (TTFA). TTFA binds to the quinone biding site on complex II, preventing ubiquinone binding and thus electron transport (35). Growth of the WT and the Δ*vibF* mutant in the presence of TTFA did not affect the activation state of the Cpx system (Fig. 6), suggesting that inhibition of complex II activity was not sufficient to activate the Cpx system in the WT or to suppress Cpx activation in JB485. In contrast, TTFA activated the Cpx system in a *vibF*-independent manner in JB485. This was evidenced by the fact that JB485 produced dark blue colonies and JB485Δ*vibF* produced diffuse light blue colonies on the TTFA plates (Fig. 6). We cannot explain the RND efflux-dependent effects of TTFA on the Cpx system. It is possible that TTFA is a substrate for the RND transporters. This would result in increased TTFA uptake in JB485, which could serve to activate the Cpx system through an ROS-specific pathway, as electrons are unable to be efficiently passed from complex II. Alternatively, the absence of RND-mediated efflux may result in pleiotropic effects on the Cpx system in JB485 that are compounded by TTFA.

## DISCUSSION

In this report, we have expanded on the function of the *V. cholerae* RND efflux systems by demonstrating that the VexGH RND transporter contributes to vibriobactin export. As discussed below, our results provide the first evidence to link RND-mediated efflux to iron acquisition and the maintenance of cellular homeostasis in *V. cholerae* and provide insight into the selective pressures for the maintenance of what was previously thought to be a redundant RND transporter.

Our results demonstrate that the VexGH RND efflux system functions in vibriobactin export. This conclusion was supported by several lines of evidence, including the fact that there were reduced amounts of vibriobactin present in culture supernatants of mutants lacking *vexH* (Fig. 2) and that vibriobactin export was impaired in strains lacking RND-mediated transport (Fig. 3). Iron is a cofactor for many biological processes and is therefore an essential nutrient for nearly all life forms (36). Vibriobactin is produced by *V. cholerae* as a mechanism to acquire environmental iron (12). Vibriobactin is synthesized in the cytoplasm before being secreted. In many Gram-negative bacteria, siderophore secretion appears to occur by a two-step process where the siderophore is first translocated into the periplasm before being secreted into the external environment. Once outside the cell, siderophores bind to ferric iron before being taken back up into the cell via specific transporters, delivering the iron payload to the cytoplasm for use in metabolism. Although the processes of vibriobactin biosynthesis and uptake are well understood (37, 38), the mechanism by which vibriobactin is secreted into the environment is unknown. Our results strongly suggest that VexGH contributes to this process.

The finding that a *vexGH* mutant phenocopied an RND null mutant for vibriobactin secretion (Fig. 3) suggested that the VexGH RND system may be the primary RND transporter involved in vibriobactin secretion. This conclusion is bolstered by the finding that *vexGH* is coregulated with the vibriobactin biosynthetic genes in response to iron (24). However, we cannot completely exclude the possibility that other RND transporters contribute to vibriobactin secretion. Other transporters may have been missed because of the sensitivity of the assay. It is also possible that the mutation of individual RND transporters effects increased expression of redundant transporters, which could mask the phenotype of specific RND mutants in vibriobactin export. In addition to being regulated by Fur, *vexGH* is also positively regulated by the Cpx system in response to cell envelope perturbations (9, 11). This suggests that the function of VexGH extends beyond vibriobactin secretion to protecting the cell from deleterious effects of toxic environmental compounds. The latter function is consistent with previous studies showing that VexGH is a multidrug efflux system that provides resistance to bile acids, nonionic detergents, ampicillin, and novobiocin (8).

All six *V. cholerae* RND transporters were found to be important for virulence factor production (7). The expression of *vexAB* was upregulated in human- and animal-shed *V. cholerae*, while that of *vexGH* and *vexIJK* was induced *in vivo* in human volunteers (7, 39, 40). These collective studies highlight the importance of the RND transporters in pathogenesis, but the contribution of each individual system to pathogenesis was unclear. We previously showed that four of the *V. cholerae* RND transporters had overlapping substrate specificity for bile, an important barrier to colonization (7, 8). VexB, VexD, VexH, and VexK functioned in bile salt resistance, with VexB and VexD being major contributors relative to VexH and VexK, which provided modest but equivalent contributions to bile salt resistance (8). Interestingly, while the antimicrobial susceptibility profile of mutants lacking the VexBDH or VexBDK efflux pumps were similar, the former strain was nearly 4 log units more attenuated during single-strain colonization of the infant mouse intestine than the latter strain (8). This suggested that VexH contributed much more to intestinal colonization than VexK. The mechanism behind this is unclear, but on the basis of our results showing that VexG functions in vibriobactin secretion and that vibriobactin retention is detrimental to growth under iron-limited conditions, we suggest that the inability of the *vexH* mutant to secrete vibriobactin likely contributed to its *in vivo* attenuation. It is also possible that the inability of the *vexH* mutant to secrete vibriobactin attenuated colonization because of impaired iron acquisition. The fact that vibriobactin-negative mutants are not attenuated for infant mouse colonization argues against this possibility (41).

We previously showed that RND-mediated efflux maintained the *V. cholerae* Cpx system in a suppressed state during growth under standard laboratory conditions (9). Although the mechanism linking RND efflux to the Cpx system was unknown, we and others proposed that Cpx activation in mutants with impaired RND transport likely resulted from the intracellular accumulation of toxic metabolites that were normally removed from the cell by the RND transporters (9, 11). Our results here demonstrate that the RND-dependent toxic metabolite was vibriobactin. The inability of the RND mutant cells to secrete vibriobactin was detrimental, as evidenced by the activation of the Cpx system and the vibriobactin-dependent decreased fitness of an RND-deficient mutant during growth under iron-limited conditions. *V. cholerae* is likely to encounter iron limitation in both the host and aquatic ecosystems, which makes these results particularly relevant to its biology (42–44).

*V. cholerae*, like many Gram-negative bacteria, encodes multiple RND efflux systems with overlapping substrate specificities. Yet the selective pressures maintaining these redundant systems in the *V. cholerae* genome are unclear. The fact that vibriobactin retention was detrimental to the cell could provide the selective pressure for the maintenance of *vexGH* in members of the family *Vibrionaceae*. The detrimental effects of vibriobactin retention could also explain our finding that RND-independent mechanisms also contributed to vibriobactin export, including active transporters and porins. It is interesting to speculate that the effects of siderophore retention defined here may apply to other bacteria where efflux has been linked to Cpx activation and siderophore export (45–49). The conservation of similar findings between distantly related genera suggests that our results extend beyond *V. cholerae*.

**FIG 7:**
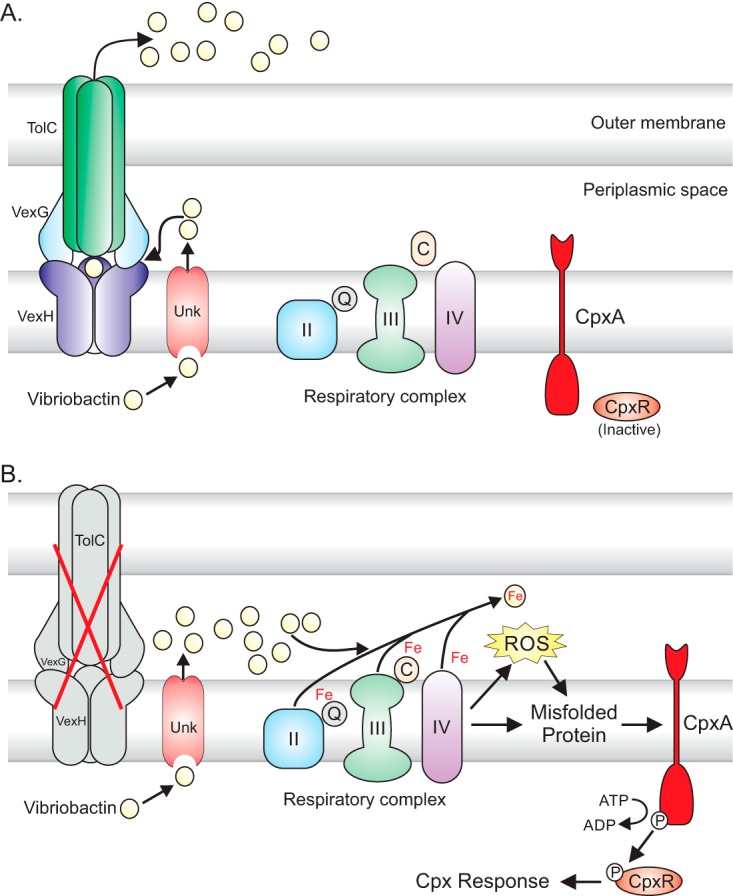
Putative model of Cpx system activation in a *vexGH* mutant. (A) In WT cells, vibriobactin is produced and exported into the periplasmic space by an unknown transporter. Periplasmic vibriobactin is then secreted into the external environment by the VexGH-TolC RND efflux system, and the CpxRA system is inactive. (B) In the absence of *vexGH*, iron-free vibriobactin accumulates in the periplasmic space and chelates iron from the iron-rich, membrane-bound components of the respiratory chain. Chelation of iron from the respiratory chain components leads to altered protein structures and/or increased ROS production, both of which can activate the Cpx system. Unk, unknown vibriobactin transporter; II, complex II; III, complex III; IV, complex IV; C, cytochrome C; P, phosphate.

Although we were unable to identify the precise mechanism by which vibriobactin activated the Cpx system in the *vexGH* mutant, the fact that complex II mutations and anaerobic growth suppressed the Cpx system suggested that the activating signal likely involved a toxic byproduct of aerobic respiration, most likely ROS, which can attack protein disulfide bonds, resulting in aberrant bond formation, a known Cpx-inducing signal. Our results showing that the Cpx system was induced upon exposure to oxidizing agents (e.g., CuCl_2_ and paraquat) support this idea. On the basis of these observations, we propose a model (Fig. 7) whereby, in the absence of RND-mediated efflux, iron-free vibriobactin accumulates within the cell and chelates iron from the iron-rich components of the ETC. The removal of iron from ETC components then generates the Cpx-inducing cue in one of two ways. The chelation of iron from the Fe-S centers in complex II (or other ETC components) could directly result in the formation of abnormal disulfide bonds in the deferrated proteins. Alternatively, the chelation of iron from ETC components could result in increased ROS production, which could then catalyze the formation of nonnative disulfide bonds in periplasmic proteins (50). We note that these two scenarios are not mutually exclusive and it is possible that combinations of both signals may contribute to Cpx activation. This model also explains the observation that iron addition suppressed the Cpx system in RND-deficient cells. Iron addition could suppress the Cpx response by several mechanisms, including repression of vibriobactin production (via Fur), inhibition of iron chelation from ETC components, and/or replacement of iron in deferrated ETC components. This model is consistent with recent reports showing that dipyridyl activated the Cpx response in *V. cholerae* (11) and can be extended to other organisms where efflux has been associated with activation of the Cpx response and/or the induction of stress responses (45–49, 51).

## MATERIALS AND METHODS

### Bacterial strains and culture conditions.

The bacterial strains used in this study are listed in Table 1. *E. coli* strain EC100D*pir*^+^ was used for cloning, and *E. coli* strain SM10λ*pir* was used to conjugate plasmids into *V. cholerae*. *V. cholerae* O1 El Tor strain N16961 Δ*lacZ* was used as the WT control in all experiments. Bacteria were routinely grown in LB broth or on LB agar at 37°C. Modified T medium was prepared as previously described (21). Anaerobic conditions were achieved with the BD GasPak EZ Pouch System. Antibiotics were used at the following concentrations: streptomycin, 100 µg/ml; carbenicillin, 100 µg/ml; kanamycin, 50 µg/ml.

**TABLE 1:** Strains and plasmids used in this study

Strain or plasmid	Genotype or description	Source
*E. coli*		
EC100D*pir*^+^	F^−^ *mcrA* Δ(*mrr-hsdRMS-mcrBC*) φ80d*lacZ*ΔM15 Δ*lacX74 recA1 endA1* *araD139* Δ(*ara*, *leu*)*7697 galU galK* λ^−^ *rpsL* (Str^r^) *nupG pir*^+^	Epicenter
SM10λ*pir*	*thi-1 thr leu tonA lacY supE recA*::RP4-2-4-Tc::Mu Km^r^ (λ *pir*R6K)	53
*V. cholerae*		
JB58	O1 El Tor strain N16961 Δ*lacZ* Sm^r^	Lab collection
JB485	JB58 Δ*vexB* Δ*vexD* Δ*vexF* Δ*vexH* Δ*vexK* Δ*vexM*	7
XBV247	JB58 Δ*vibF*	This work
XBV251	JB485 Δ*vibF*	This work
XBV310	JB58 Δ*vibC*	This work
XBV307	JB485 Δ*vibC*	This work
JB150	JB58 Δ*tolC*	54
JB116	JB58 Δ*vexH*	7
LSΔR	JB58 Δ*cpxR*	10
DK56	JB58Δ*vibF cpxP-lacZ*	This work
DK53	JB485Δ*vibF cpxP-lacZ*	This work
DT1460	JB485*cpxP-lacZ*	9
DT1458	JB58*cpxP-lacZ*	9
Plasmids		
pTL61T	*lacZ* transcriptional reporter, Amp^r^	55
pXB229	pTL61T containing *vexGH* promoter	9
pDK4	pTL61T containing *irgA* promoter	This work
pDK6	pTL61T containing the *vibF* promoter	This work
pDK7	pTL61T containing *hutA* promoter	This work
pWM91	Allelic exchange vector, Amp^r^	56
pWM91-Δ*vibF*	Used for deletion of *vibF*	This work
pWM91-Δ*vibC*	Used for deletion of *vibC*	This work
pBAD18	Arabinose-inducible expression vector, Amp^r^	57
pBAD18-*vibF*	*vibF* expression vector	This work

### Plasmid and mutant construction.

The plasmids used in this study are listed in Table 1; for the oligonucleotides used in this study, see Table S1. Transcriptional reporters for *irgA* (VC0475), vibF (VC2209) and *hutA* (VC0576) were constructed by cloning the promoter region of each respective gene in front of the *lacZ* gene in pTL61T. Briefly, N16961 genomic DNA was used as a template for PCR with primers P-VC0475 and F-XhoI/P-VC0475-R-BamHI. The resulting amplicon was digested with the XhoI and BamHI restriction endonucleases before being ligated into similarly digested pTL61T to generate pDK4. pDK6 (*vibF*-*lacZ*) and pDK7 (*hutA*-*lacZ*) were constructed similarly using the promoter specific PCR primers listed in Table S1. The *vibF* (VC2209) deletion construct was constructed as follows. Primers *vibF*-F1 and *vibF*-R2 and primers *vibF*-F2 and *vibF*-R1 were used in separate PCRs with N16961 genomic DNA. The resulting ~1.5-kb amplicons were collected and used as the template for second-round PCR amplification with the flanking *vibF*-F1 and *vibF*-R1 PCR primers. The resulting ~3-kb amplicon was then digested with the SpeI and SmaI restriction endonucleases before being ligated into similarly digested pWM91 to generate pWM91-Δ*vibF*. The *vibC* (VC0773) deletion construct was constructed in a similar manner with primers *vibC*-F1 and *vibC*-R2 and primers *vibC*-F2 and *vibC*-R1. Unmarked in-frame deletion of *vibC* and *vibF* in each respective strain was constructed by allelic exchange with pWM91-Δ*vibC* and pWM91-Δ*vibF* as previously described (7). The *vibF* gene was cloned into pBAD18 in a two-step cloning procedure. N16961 genomic DNA was used as a PCR template for two concurrent PCRs with primers VC2209-pBAD-F1 and VC2209-pBAD-R2 and primers VC2209-pBAD-F2 and VC2209-pBAD-R1 to produce a 2,957-bp fragment and a 4,366-bp fragment, respectively. The 2,957-bp amplicon was digested with the EcoRI and KpnI restriction endonucleases before being ligated into the similarly digested pBAD18 vector. The resulting plasmid was then digested with the KpnI and SalI restriction endonucleases and ligated with the similarly digested 4,366-bp replicon to produce pBAD18-*vibF*.

10.1128/mBio.00126-17.2 TABLE S1  Oligonucleotides used in this study. Download TABLE S1, DOCX file, 0.01 MB. Copyright © 2017 Kunkle et al. 2017 Kunkle et al. This content is distributed under the terms of the Creative Commons Attribution 4.0 International license.

### Identification of Cpx suppressors.

A mariner transposon library was generated in JB485::*cpxP-lacZ* with pNJ17 as previously described (52). The transposon library was then plated onto LB–X-Gal plates and incubated at 37°C. The following day, white colonies were selected and the transposon insertion sites in the white colonies were identified by DNA sequencing of arbitrary PCR products as previously described (52).

### Growth analysis.

Growth curves were generated in microtiter plates as follows. The strains indicated were grown overnight in T medium with FeSO_4_ supplementation. The overnight cultures were then washed once in 1 volume of phosphate-buffered saline (PBS) before being diluted 1:100 in fresh T medium plus or minus FeSO_4_. Two-hundred-microliter volumes of the diluted cultures were then placed in triplicate wells of a 96-well microtiter plate. The microtiter plates were then incubated at 37°C with constant shaking, and the OD at 600 nm (OD_600_) was measured every 30 min. The OD at each time point was averaged and plotted against time to generate the growth curves reported.

### Analysis of Cpx expression on agar plates.

A chromosomal *cpxP-lacZ* reporter was used to assess the activation state of the Cpx system as previously described (9). Briefly, overnight LB broth cultures of the test strains were inoculated into fresh LB broth and incubated with shaking for 1 h, and then the cultures were normalized to an OD_600_ of 0.1. The cells were then collected by centrifugation and resuspended in 1 volume of PBS. The cultures were then diluted 1:1,000 in PBS, and 2 μl of the diluted culture was spotted onto the surfaces of LB agar plates containing 160 μg/ml X-Gal and other additives as indicated. The inoculated plates were incubated overnight at 37°C before being photographed.

### Transcriptional reporter assays.

*V. cholerae* strains containing the reporter plasmids indicated were collected from the surfaces of LB agar plates and resuspended in LB broth to an OD_600_ of 0.6. The cultures were then used to inoculate 5 ml of fresh LB broth (1:100), which was then incubated at 37°C with shaking for 3 h, and then culture aliquots were collected in triplicate and the β-galactosidase activity was quantified (time zero). The remaining cultures were then treated by addition of the carrier and dipyridyl (130 μM), FeSO_4_ (130 μM), or both before being incubated at 37°C with shaking for an additional hour, and then aliquots were taken in triplicate for the β-galactosidase assay. The experiments were performed independently at least three times, and β-galactosidase production was calculated and displayed in Miller units.

### CAS assays.

CAS assays were performed as previously described (20). Briefly, the test strains were cultured in T medium without FeSO_4_ supplementation at 37°C with shaking for 18 h. Cleared culture supernatants were then generated by centrifugation before triplicate 100-μl aliquots of the supernatant were collected from each strain. The amount of siderophore in the supernatants was then assessed by mixing 100 μl of modified CAS assay solution with 100 μl of culture supernatant in a 96-well microtiter plate. The solutions were then allowed to equilibrate for 3 h, and then the absorbance at 630 nm was measured with a Biotek Synergy 4 microplate reader. Total siderophore production was assessed by using 18 h cultures of the strains indicated that had been subjected to five freeze-thaw cycles. The resulting cell lysate were then used in the CAS assay as described above.

### Cross-feeding growth stimulation assays.

Cross-feeding growth stimulation bioassays were performed as previously described (22). Briefly, the growth indicator plates were prepared by inoculating 10 μl of an overnight saturated culture of the *V. cholerae* Δ*vibC* mutant indicator strain into 20 ml of ~50°C LB agar containing 270 μM dipyridyl. The inoculated agar was then poured into 100-mm petri plates and allowed to solidify. The use of 270 μM dipyridyl in the LB agar was empirically determined to inhibit the growth of the Δ*vibC* mutant indicator strain. The solidified agar was then inoculated with the test strains by spotting 5-μl aliquots of fresh overnight broth cultures in triplicate onto the surfaces of the indicator plates. After drying, the plates were incubated at 37°C for 24 h, and then the diameter of the zone of growth of the Δ*vibC* mutant indicator strain was measured.
