# Supplementary material for: The Vibrio cholerae VexGH RND Efflux System Maintains Cellular Homeostasis by Effluxing Vibriobactin
Source: mBio. 2017 May 16;8(3):e00126-17. doi: 10.1128/mBio.00126-17 (PMC5433094; doi:10.1128/mBio.00126-17)
Supplement: TABLE S1 [file mbo003173311st1.docx]

| **Table S1. Oligonucleotides used in this study.** | | |
| --- | --- | --- |
| **Oligonucleotides:** | **Sequence (5’-3’):** |  |
| P_VC0475_-F-XhoI | AACTCGAGAGACGTTCCATATTTGGACCG |  |
| P-_VC0475_-R-BamHI | GGGGATCCAAACATTAAGCCTAGTGTCACAG |  |
| P-_VCA0576-R-BamHI_ | TTGGATCCGATCGCGCTAGAGAGCAGAG |  |
| P-_VCA0576-F-XhoI_ | AACTCGAGCCGCCAACTGTGATGAGAGTC |  |
| *vibF*-F1-SmaI | TACCCGGGACCAACTTAAACAGCAGCCTCGC |  |
| *vibF*-F2 | GCGGCTTATTGGGAAGATGAAGCTTGTCGCTCTCCGTCCATG |  |
| *vibF*-R1-SpeI | CCACTAGTCATTCTCTTCATCTCCAGATATAC |  |
| *vibF*-R2 | AGCTTCATCTTCCCAATAAGCCGCTTGCATTGCTGTCATTTC |  |
| VibC-F1-BamHI | GCGGGATCCCCAACCGAAATTGCGCAGC |  |
| VibC-F2 | GGTCGGATACACCTCAACGATGTGCTCACCGCTTAAGGAG |  |
| VibC-R-SacI | GCGAGCTCCAGAGTAGTGCTAAAGGCGGCACCAAC |  |
| VibC-R2 | GCACATCGTTGAGGTGTATCCGACCACTTCACGCTTCATGACC |  |
| VC2209-pBAD-F | ATGAATTCTCTTTAAAGATGAAAGAAA |  |
| VC2209-pBAD-R | ATGTCGACCTACCCCATGGACGGAGA |  |
| VC2209-pBAD-F2 | GTTTGGCTTCTGCACTTTCGGGG |  |
| VC2209-pBAD-R2 | TGGGAGACGCAGCATCAATAGG |  |
